# Bias-corrected NESM3 global dataset for dynamical downscaling under 1.5 °C and 2 °C global warming scenarios

**DOI:** 10.1038/s41597-024-03224-0

**Published:** 2024-04-20

**Authors:** Meng-Zhuo Zhang, Ying Han, Zhongfeng Xu, Weidong Guo

**Affiliations:** 1 School of Atmospheric Sciences, Nanjing University, Nanjing, China; 2 CAS Key Laboratory of Regional Climate and Environment for Temperate East Asia, Institute of Atmospheric Physics, Chinese Academy of Sciences, Beijing, 100029 China

**Keywords:** Projection and prediction, Hydrology, Environmental impact

## Abstract

Dynamical downscaling is vital for generating finer-scale climate projections. Recently, a set of simulations under four types of 1.5/2 °C global warming scenarios are available with Nanjing University of Information Science and Technology Earth System Model (NESM). However, NESM3’s bias in large-scale driving variables would degrade downscaled simulations. We corrected NESM3 bias in terms of climate mean and inter-annual variance against ERA5 using a novel bias correction method and then produced a set of bias-corrected datasets for dynamical downscaling. The bias-corrected NESM3 spans the historical period for 1979–2014 and four future scenarios (i.e., 1.5 °C overshoot for 2070–2100, stabilized 1.5/2 °C for 2070–2100, and transient 2 °C for 2031–2061) with 1.25° × 1.25° horizontal resolution at six-hourly intervals. Our evaluation suggests that bias-corrected NESM3 outperforms the original NESM3 in the climatological mean of seasonal mean and variability, as well as climate extreme events during the historical period. This bias-corrected dataset is expected to generate more reliable projections for regional climate and environment under 1.5/2 °C global warming.

## Background & Summary

Climate change tends to cause sea level rise and more frequent climate extremes, such as heatwaves and heavy precipitation, resulting in serious damage to human society and ecosystems^1,2^. To avoid irreversible climate change, the Paris Agreement in 2015 aimed to limit the increase in global mean surface temperature (GMST) below 2 °C and preferably keep the rise below 1.5 °C above the preindustrial level^3,4^. Since then, climate change and its impact under 1.5 and 2 °C warming have received increasing attention^5–8^.

The general circulation model (GCM) is a primary tool to investigate climate change under 1.5 and 2 °C warming scenarios. Note that in this paper, GCM refers to the atmosphere-ocean coupled general circulation model or earth system model for simplicity. Many studies used GCM simulations derived from the Coupled Model Inter-comparison Project (CMIP) under Representative Concentration Pathways or Shared Socioeconomic Pathways to examine the climate change in the transient 1.5 and 2 °C warmer GMST^9–11^. As the increase in GMST to 1.5 and 2 °C presents transiently in these simulations, CMIP GCMs cannot deal with the climate change under stabilized 1.5 and 2 °C global warming^9,12–14^, limiting the knowledge of the stabilized 1.5 and 2 °C warmer worlds. To fill the gap, a set of ensemble simulations using the Community Earth System Model (CESM) were produced under three low-warming scenarios in line with the Paris targets, including the stabilization pathways at 1.5 and 2 °C global warming^12^, which are widely used in many studies^15–17^. Recently, four types of ensemble experiments on 1.5 and 2 °C global warming scenarios were carried out using the Nanjing University of Information Science and Technology Earth System Model (NESM), i.e., 1.5 °C overshoot, stabilized 1.5 °C, stabilized 2 °C, and transient 2 °C^18^. Compared with the CESM low-warming simulations, NESM3 additionally carried out a simulation under a transient 2 °C global warming scenario, which can provide a more comprehensive understanding of the 1.5 and 2 °C warmer worlds.

However, with a coarse horizontal resolution of 1.25° × 1.25°, the NESM3 cannot provide fine-scale climate change information under 1.5 and 2 °C warmer worlds for many climate-related studies, such as vulnerability, impact, and adaptation assessment and climate extreme projections. Dynamical downscaling is one of the widely-used approaches to generating spatially refined regional climate simulations. The traditional dynamical downscaling approach uses the GCM simulations as the initial and lateral boundary conditions to drive the regional climate model (RCM)^19–21^. As the bias in the initial and lateral boundary conditions can propagate into the RCM, the accuracy of downscaling simulations is largely dependent on the quality of GCMs’ output^22–26^. Like other GCMs, NESM3 also suffers from systematic bias in terms of various driving variables during the historical period^27^. Therefore, it is necessary to correct the NESM3 bias to provide high-quality large-scale driving fields for dynamical downscaling projections under the 1.5 and 2 °C global warming scenarios.

Over the past decade, many GCM bias correction methods have been developed, e.g., mean bias correction^28^, mean and variance bias corrections^29,30^, quantile-quantile correction^31,32^, and multi-model ensemble bias correction^33^. Recently, Xu *et al*.^34^ proposed a novel mean-variance-trend (MVT) method, which integrates the advantages of some previous GCM bias correction methods. Our evaluation suggested that the MVT method enables to improve dynamical downscaling simulations of multiple variables in terms of their climatological mean, interannual-to-interdecadal variances, annual cycle, and day-to-day variability^35^. Therefore, with the aid of the MVT method, we corrected the climatological mean and inter-annual variance biases in NESM3 based on the European Centre for Medium-Range Weather Forecasts Reanalysis 5 (ERA5) dataset. The bias corrections were applied to historical simulation during 1979–2014 and four types of future simulations on 1.5/2 °C global warming scenarios. This bias-corrected dataset provides high-quality large-scale driving fields for dynamical downscaling under 1.5 and 2 °C global warming scenarios, which is expected to generate more reliable simulations at the regional scale relative to the dynamical downscaling using raw GCM data.

## Methods

### Data acquisition

We used the six-hourly data of the first ensemble run produced by the NESM3 during the historical and future periods. The historical simulation during 1979–2014 is available in the CMIP Phase 6 (CMIP6) historical experiments^36–38^. The future simulations include four types of the 1.5 and 2 °C global warming scenarios, i.e., the stabilized 1.5 °C scenario during 2070–2100, the 1.5 °C overshoot scenario during 2070–2100, the stabilized 2 °C scenario during 2070–2100, and the transient 2 °C scenario during 2031–2061^18,39^. For the stabilized 1.5 and 2 °C scenario, the GMST warming is designed to be stabilized at the level of 1.5 and 2 °C ( ± 0.1 °C) above preindustrial levels for at least three decades by 2100, respectively. The 1.5 °C overshoot scenario is designed such that the GMST warming slightly overshoots before returning to the 1.5 °C by 2100. For the transient 2 °C scenario, the GMST is designed to continuously increase and reach 2 °C by 2050. These four types of future simulations can help to comprehensively assess the climate impacts of 1.5 and 2 °C global warming and understand the driving mechanism of climate change in different warming scenarios. Compared with the CESM low-warming simulations, the additional scenario of the transient 2 °C can also be used to assess the difference and similarity of climate responses under transient and stabilized global warming. Besides, the NESM3 is configured with a horizontal resolution of 1.9° by 1.9° in the atmosphere and 1° by 1° in the ocean^37^. The used variables include the five upper air variables (i.e., air temperature, geopotential height, specific humidity, zonal wind, and meridional wind) and six surface variables (i.e., surface pressure, sea level pressure, sea surface temperature, soil moisture, soil temperature, and 2 m temperature). Therein, upper air variables are at 17 different pressure levels, including 1000, 925, 850, 700, 600, 500, 400, 300, 250, 200, 150, 100, 70, 50, 30, 20, and 10hPa. Soil moisture and temperature consist of 4 depth levels, including 0.05, 0.25, 0.7, and 1.5 m.

We also used the high-resolution reanalysis dataset—ERA5 during 1979–2014^40,41^. Assimilating vast amounts of observations by advanced modeling and data assimilation system, ERA5 shows improvements compared with the ERA-Interim in many aspects, e.g., cloud, precipitation, wind fields, and humidity^42–44^. Both the NESM3 and ERA5 are re-gridded to the horizontal resolution of 1.25° by 1.25° using the bilinear interpolation with the aid of climate data operator (CDO).

### GCM bias-correction method

Similar to the MVT method proposed by Xu *et al*.^34^, we corrected the NESM3 biases of the inter-annual variance and climate mean. As the future simulations under 1.5 and 2 °C global warming scenarios are not available with a limited number of CMIP6 models, we did not adjust the long-term non-linear trend of NESM3 using the trend derived from the multi-model ensemble mean. Instead, we preserved the long-term non-linear trend of NESM3.

The bias corrections were applied to the six-hourly data. For each six-hour period and day of the year, the multi-year time series derived from the NESM3 simulation and the ERA reanalysis can be broken down into a long-term non-linear trend plus an inter-annual perturbation term, respectively:

1 $${\rm{N}}{\rm{E}}{\rm{S}}{\rm{M}}={{\rm{N}}{\rm{E}}{\rm{S}}{\rm{M}}}_{LT}+{{\rm{N}}{\rm{E}}{\rm{S}}{\rm{M}}}^{{\rm{{\prime} }}}$$

2 $${\rm{E}}{\rm{R}}{\rm{A}}={{\rm{E}}{\rm{R}}{\rm{A}}}_{LT}+{{\rm{E}}{\rm{R}}{\rm{A}}}^{{\rm{{\prime} }}}$$

The non-linear trend was calculated using the monthly mean data by the ensemble empirical mode decomposition (EEMD) method^45^. EEMD is an intuitive, direct, and adaptive method based on empirical mode decomposition, which can extract coexisting oscillations of different frequencies in the original data to a few separate components^45^. Here, the non-linear trend is a monotonic curve that filters all high-frequency fluctuation.

We first corrected the variance bias of NESM3 by multiplying a scaling factor derived from the inter-annual perturbation term:

3 $${{\rm{N}}{\rm{E}}{\rm{S}}{\rm{M}}}_{v}={{\rm{N}}{\rm{E}}{\rm{S}}{\rm{M}}}_{LT}+{{\rm{N}}{\rm{E}}{\rm{S}}{\rm{M}}}^{{\rm{{\prime} }}}\times {r}_{s}^{hist}$$

4 $${r}_{s}^{hist}=\frac{{\sigma }_{{\rm{ERA}}}}{{\sigma }_{{\rm{NESM}}}^{hist}}$$

where ${r}_{s}^{hist}$ is the ratio of the standard deviation of the detrended ERA5 reanalysis to that of the detrended NESM3 simulation during the historical period (1979–2014), and NESM_*v*_ is the NESM3 simulation with bias-corrected variance. We calculated the standard deviation using all 36 years of data. Then, we removed the years with anomalies greater than three times the original standard deviation and recalculated the standard deviation to remove the unrealistic standard deviation induced by extreme weather systems, e.g. tropical cyclones. Note that the bias correction of the inter-annual variance for each six-hour of the year is conducted on the detrended NESM3, which cannot completely reduce the bias of the inter-annual variability of NESM3 because the inter-annual variance of the long-term trend is not considered here.

Next, we removed the climatological mean bias of NESM3 for each six-hour of the year. Since the climatological mean value is included in the long-term non-linear trend at the monthly scale, we replaced the climatological mean values of the non-linear trend $\overline{({{\rm{NESM}}}_{LT}}$) with the climatological mean values for each six-hour of the year ($\overline{{\rm{NESM}}}$) in the NESM3. Subsequently, the bias-corrected data can be constructed as follows:

5 $${{\rm{N}}{\rm{E}}{\rm{S}}{\rm{M}}}_{mv}={{\rm{N}}{\rm{E}}{\rm{S}}{\rm{M}}}_{LT}-\bar{{{\rm{N}}{\rm{E}}{\rm{S}}{\rm{M}}}_{LT}}+\bar{{\rm{N}}{\rm{E}}{\rm{S}}{\rm{M}}}-\bar{({{\rm{N}}{\rm{E}}{\rm{S}}{\rm{M}}}^{hist}}-\bar{{\rm{E}}{\rm{R}}{\rm{A}}5})+{{\rm{N}}{\rm{E}}{\rm{S}}{\rm{M}}}^{{\rm{{\prime} }}}\times {r}_{s}^{hist}$$

where the overbar indicates the climatological mean, and $\bar{({{\rm{N}}{\rm{E}}{\rm{S}}{\rm{M}}}^{hist}}-\bar{{\rm{E}}{\rm{R}}{\rm{A}}5})$ is the climatological mean bias of NESM3 for each six-hour of the year during the historical period. Thus, the bias-corrected six-hourly NESM3 data (NESM_*mv*_) preserved the long-term non-linear trend and removed the biases of climate mean and inter-annual variance. Under the assumption that the historical biases remain stationary in future scenarios, the bias corrections were conducted to the NESM3 historical and future simulations according to the NESM3 biases during historical period. Besides, considering the significant difference in the inter-annual variance between the sea water and sea ice surface, we did not correct the variance bias of the sea surface temperature in the NESM3 for the region where there is sea ice (water) in the NESM3 while sea water (ice) in the ERA5 during historical period.

### Evaluation method

For the time series, we evaluated the bias-corrected NESM3 performance by the mean and standard deviation. Besides, the frequency distribution of the time series was also taken into account, and we used the Kolmogorov–Smirnov statistic to evaluate the goodness-of-fit^46^. A larger value for the Kolmogorov–Smirnov statistic represents more difference between the two distributions^46^.

To give a more comprehensive evaluation, we focused on the spatial fields of the seasonal mean and variability as well as the extreme values simulated by the bias-corrected NESM3. Here, the 2nd percentile and 98th percentile of daily simulations were used as the extreme values, following the framework of VALUE^47^. Four centered statistics were used to measure model performance in terms of the scalar fields, i.e., mean error (ME), standard deviation (SD), correlation coefficient (CORR), and root mean square error (RMSE). Similarly, we also used the vector mean error (VME), the centered root mean square length (cRMSL), the centered vector similarity coefficient (cVSC), and the root mean square vector difference (cRMSVD)^48–50^ for the vector fields, such as wind fields. Note that these four statistics take the wind speed and wind direction into consideration simultaneously^48–50^. ME (VME) and RMSE (RMSVE) measure the mean bias and the overall bias for a scalar (vector) variable, respectively. SD (cRMSL) measures the amplitude of an anomalous scalar (vector) field, and CORR (cVSC) measures the pattern similarity between two anomalous scalar (vector) fields.

## Data Records

The dataset presented in this paper was open access in the China Science Data Bank^51^. The NetCDF data were compressed to save space. Users should use two attributes (i.e., scale_factor and add_offset) to unpack the variables. We also provide a FORTRAN code to convert these compressed NetCDF files to WRF intermediate files. The dataset includes three surface variables and five upper air variables for five sets of bias-corrected NESM3 simulations at six-hourly interval, i.e., the historical data from 1979 to 2014, the transient 2 °C scenario from 2031 to 2061, as well as the stabilized 1.5 °C scenario, the 1.5 °C overshoot scenario, and the stabilized 2 °C scenario from 2070 to 2100 (Table 1). The upper air variables consist of 17 pressure levels (1000, 925, 850, 700, 600, 500, 400, 300, 250, 200, 150, 100, 70, 50, 30, 20, and 10hPa). In addition, the soil moisture, the soil temperature, and the 2 m temperature are also provided as the initial conditions (ICs) for the dynamical downscaling. Considering that the impact of the ICs is neglectable for long-term RCM simulations, the three variables are at the monthly scale. Due to the missing data for the soil moisture and the soil temperature of NESM3 during the historical period, we used the ERA5 data as the historical soil conditions and provided the future soil conditions from the NESM3 with no bias correction. Note that the monthly 2 m temperature is bias-corrected in terms of the climate mean during both historical and future periods, which is used as the surface temperature of the land when generating the ICs for RCM.

**Table 1:** Variables in the bias-corrected NESM3 dataset. The tick represents the NESM3 simulation of a variable with bias corrections. ‘ERA5’ represents the simulation of a variable derived from the ERA5, while ‘Raw’ indicates the original NESM3 simulation. The variable with the star symbol is provided at a monthly time scale, and the others are at six-hourly interval.

Variables	Acronym	Vertical levels	Historical (1979–2014)	Transient 2 °C (2031–2061)	Stabilized 1.5 °C (2070–2100)	1.5 °C overshoot (2070–2100)	Stabilized 2.0 °C (2070–2100)
Surface pressure	ps	1	√	√	√	√	√
Sea level pressure	psl	1	√	√	√	√	√
Sea surface temperature	tos	1	√	√	√	√	√
Zonal wind	ua	17	√	√	√	√	√
Meridional wind	va	17	√	√	√	√	√
Air temperature	ta	17	√	√	√	√	√
Specific humidity	hur	17	√	√	√	√	√
Geopotential height	zg	17	√	√	√	√	√
Soil moisture*	mrsol	4	ERA5	Raw	Raw	Raw	Raw
Soil temperature*	tsl	4	ERA5	Raw	Raw	Raw	Raw
2 m temperature*	tas	1	√	√	√	√	√

The horizontal resolution of the dataset is 1.25° by 1.25°. All the bias-corrected data were stored in a self-describing NetCDF format. The NetCDF files are named “atm_experiment_yyyy_mm.nc”, where “experiment” donates different experiments, i.e., hist = historical, 1.5_overshoot = 1.5 °C overshoot scenario, 1.5_stable = stabilized 1.5 °C scenario, 2.0_stable = stabilized 2 °C scenario, and 2.0_transient = transient 2 °C scenario. “yyyy” and “mm” donate the year and the month of the data, respectively. Similarly, the monthly dataset of the soil moisture, the soil temperature, and the 2 m temperature are stored in the NetCDF files named “lnd_experiment_yyyy_mm.nc”. Each NetCDF file includes all the six-hourly or monthly data for one month of a year. The complete dataset is about 1.57TB in size. All the data are freely available at the China Science Data Bank^51^.

## Technical Validation

As a state-of-the-art reanalysis dataset, the ERA5 reanalysis is regarded as the reference data to correct the NESM3 historical and future simulations. To validate the bias-corrected data, we compared the performance of the original and the bias-corrected NESM3 during the historical period against the ERA5. The difference between the bias-corrected NESM3 and the ERA5 represents the remaining bias that was not corrected. Compared with the NESM3, a smaller bias in the bias-corrected NESM3 indicates that the MVT bias correction is more effective, and vice versa.

### Time series

**Fig. 1:**
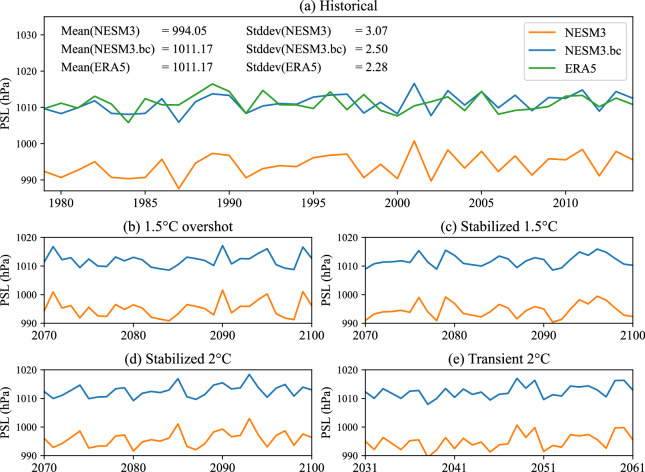
Time series of sea level pressure (PSL) in the Himalalys (30°N, 82.5°E) at 0900 UTC on July 15 in (**a**) historical and (**b,c,d,e**) future simulations. During historical period (1979–2014), the NESM3 data and the bias-corrected NESM3 data (NESM3.bc) are compared against the ERA5 data. The 36-year averaged value (Mean) and temporal standard deviation (Stddev) for each dataset are shown in the upper-left of the panel (**a**). During future periods, the time series for the NESM3 and NESM3.bc are shown in the (**b**) 1.5 °C overshoot scenario (2070–2100), (**c**) stabilized 1.5 °C scenario (2070–2100), (**d**) stabilized 2 °C scenario (2070–2100), and (**e**) transient 2 °C scenario (2031–2061).

To explicitly illustrate the changes induced by the GCM bias correction, we used the sea level pressure (PSL) in the Himalayas (30°N, 82.5°E) as an example. The time series of the original and the bias-corrected NESM3 are compared with that of the ERA5 data during the historical period (Fig. 1a). The NESM3 significantly underestimates the climatological mean of PSL by 17.12hPa against the ERA5 (0.05 significance level of student-t test), while the climatological mean of PSL in the bias-corrected NESM3 is the same as the ERA5. Regarding the inter-annual standard deviation, there is a significant overestimation in the NESM3 relative to the ERA5 (0.1 significance level of F-test), characterized by 3.07hPa in the NESM3 against 2.28hPa in the ERA5. With the standard deviation of 2.5hPa, the bias of the inter-annual variability is largely removed in the bias-corrected NESM3. Due to no bias correction for the inter-annual variance of the long-term trend, the bias of the inter-annual variability in the raw NESM3 is not completely reduced in the bias-corrected NESM3. Similar to the historical period, the bias-corrected NESM3 shows a greater climatological mean and smaller inter-annual variability than the raw NESM3 in the four scenarios during the future periods (Fig. 1b–e). Note that the bias correction method affects the climatological mean and inter-annual variability of the time series but does not alter the phase in the time series.

**Fig. 2:**
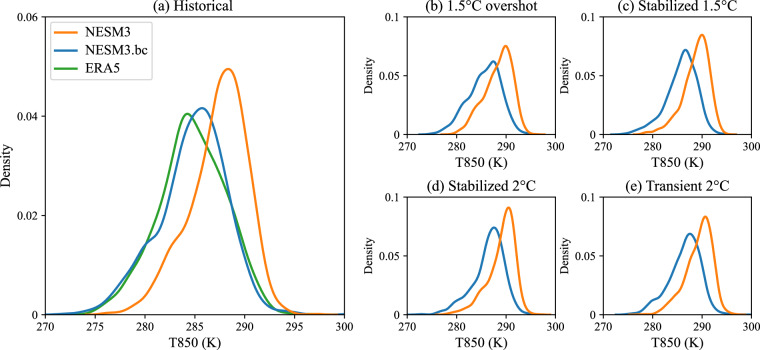
Frequency distribution of 850hPa air temperature (T850) in the western North Pacific (45°N, 150°E) at 0300 UTC in July in (**a**) historical and (**b,c,d,e**) future simulations. During historical period (1979–2014), the NESM3 data and the bias-corrected NESM3 (NESM3.bc) data are compared against the ERA5 data, and the curve of each data contains a total of 1116 days (36 years × 31 days). During future periods, the frequency distribution of the NESM3 and NESM3.bc are shown in the (**b**) 1.5 °C overshoot scenario (2070–2100), (**c**) stabilized 1.5 °C scenario (2070–2100), (**d**) stabilized 2 °C scenario (2070–2100), and (**e**) transient 2 °C scenario (2031–2061). A total of 961 days (31 years × 31 days) are included in the curve of each data in the future scenario.

Besides, the frequency distribution of 850hPa air temperature (T850) over the western North Pacific (45°N, 150°E) was also evaluated in the original and the bias-corrected NESM3 (Fig. 2). During the historical period, there is an obvious overestimation of the high-temperature events between 287–295 K in the NESM3 (Fig. 2a). With the Kolmogorov–Smirnov statistic of 0.36, the NSEM3 shows significantly different frequency distribution against that for the ERA5 (0.05 significance level of Kolmogorov–Smirnov test). By contrast, the bias-corrected NESM3 shows no significant difference in frequency distribution with that of the ERA5 (0.05 significance level of Kolmogorov–Smirnov test), characterized by the Kolmogorov–Smirnov statistic of 0.05 (Fig. 2a). Generally, the frequency distribution in the bias-corrected NESM3 has higher goodness-of-fit than that for the original one. It indicates that the bias correction method can indirectly correct the frequency characteristics of the driving variable, which favors a more accurate simulation of climate extreme events. During future periods, the frequency distribution also shifts to the low temperature in the bias-corrected NESM3 relative to that for the NESM3 under each global warming scenario (Fig. 2b–e).

### Seasonal mean

**Fig. 3:**
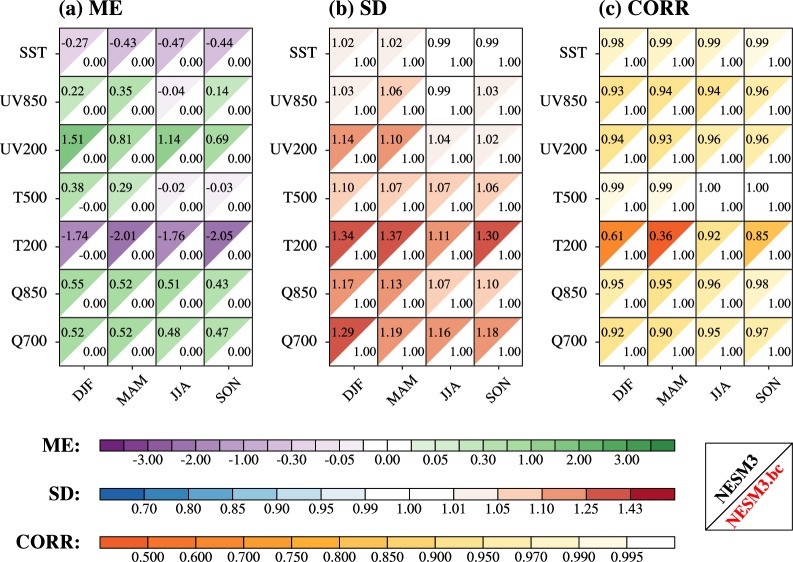
Statistical metrics to measure the performance of the NESM3 and the bias-corrected NESM3 (NESM3.bc) in simulating the climatological mean (1979–2014) of multiple driving variables in terms of the seasonal mean. Four seasons include December–January–February (DJF), March–April–May (MAM), June–July–August (JJA), and September–October–November (SON). Driving variables include sea surface temperature (SST, unit: °C), 850hPa vector winds (UV850, unit: m/s), 200hPa vector winds (UV200, unit: m/s), 500hPa air temperature (T500, unit: °C), 200hPa air temperature (T200, unit: °C), 850hPa specific humidity (Q850, unit: g/kg), and 700hPa specific humidity (Q700, unit: g/kg). The ERA5 data is used as the reference. (**a**) ME is the mean error of the GCM data against the ERA5 data, (**b**) SD is the ratio of the spatial standard deviation of the GCM data to the ERA5 data, (**c**) CORR is the spatial correlation coefficient between the GCM data and ERA5 data. Note that UV850 and UV200 are treated as vector variables in the evaluation, and then ME, SD, and CORR for the vector wind are equivalent to VME, cRMSL, and cVSC, respectively. The left-upper (right-lower) triangle in each square represents the original (bias-corrected) NESM3 performance. The color filling in each triangle donates the value of the metrics, and lighter color indicates better performance.

The performances of the original and the bias-corrected NESM3 in simulating the seasonal mean of seven driving variables in terms of the climatological mean during the historical period (1979–2014) were evaluated using ME (VME), SD (cRMSL), and CORR (cVSC) against the ERA5 data (Fig. 3). The seven driving variables can be regarded as a proxy for the CMIP6 models’ quality as driving fields for dynamical downscaling^27^. Results show that the NESM3 suffers from obvious bias in some variables, like other CMIP6 models^27^. There is a cold bias in the NESM3 simulation of global mean sea surface temperature (SST) throughout the year, especially in June–July–August (JJA) (Fig. 3a). In terms of the wind fields, the NESM3 shows inferior performance in the upper level to that of the lower level, especially during December–January–February (DJF) and March–April–May (MAM), characterized by overestimation of the mean and amplitude (Fig. 3a and b). Regarding the air temperature, the NESM3 also shows poor performance in the upper level during all seasons, which is similar to other CMIP6 models^34^. Notably, the NESM3 suffers from a cold bias in the global mean of 200hPa air temperature (T200) throughout the year (Fig. 3a). Simultaneously, the NESM3 shows the overestimated amplitude in the anomalous fields of T200 during DJF, MAM, and September–October–November (SON), along with a poor pattern similarity with the ERA5 data (Fig. 3b and c). Besides, the NESM3 shows a wet bias in the global mean of the low-level specific humidity and obviously overestimates its amplitude throughout the year (Fig. 3a and b). Compared with the original NESM3, the bias-corrected NESM3 removes all biases of the seasonal mean fields in terms of spatial mean, amplitude, and pattern similarity.

**Fig. 4:**
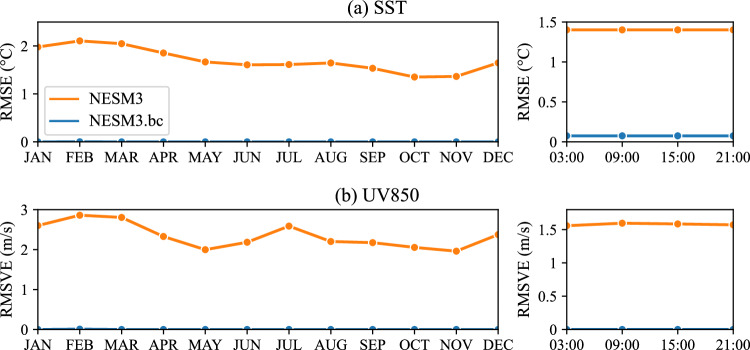
Overall bias of climatological mean fields on the monthly (left) and six-hour (right) time scales for sea surface temperature (SST) and 850hPa wind fields (UV850) in the NESM3 and the bias-corrected NESM3 (NESM3.bc). The overall bias is measured by the root mean square error (RMSE) for SST while the root mean square vector difference (RMSVE) for UV850.

In addition to the seasonal mean, the climatological mean bias on the monthly and six-hour time scales should be completely removed in the bias-corrected NESM3 against the original NESM3, according to the process of the bias corrections. Thus, we compared the overall bias of the SST and 850hPa wind fields (UV850) on the time scales of the month and six-hour in two simulations (Fig. 4). For the climatological mean of monthly SST, the NESM3 suffers from the overall bias greater than 1.35 °C, and the relatively large RMSE appears in February and March (Fig. 4a left). In terms of UV850, the NESM3 shows the RMSVE of 1.96–2.86 m/s, with relatively poor performance in February and March (Fig. 4b left). Compared with the NESM3, the bias-corrected NESM3 reduces almost all the bias of the SST and UV850. As for the climatological mean fields on six-hour time scale, the NESM3 generally shows the RMSE of approximately 1.4 °C in SST and RMSVE of 1.56–1.6 m/s in UV850 (Fig. 4 right). Compared with the NESM3, the RMSE of SST is greatly reduced to 0.08 °C, and the RMSVE of UV850 is completely removed in the bias-corrected NESM3. Note that we only corrected SST of the NESM3 in the region where there is ice or water in both NESM3 and ERA5 and kept that in the region where there is ice (water) in the NESM3 while water (ice) in the ERA5, considering the different inter-annual variance between the ice and water surface.

### Seasonal variability

**Fig. 5:**
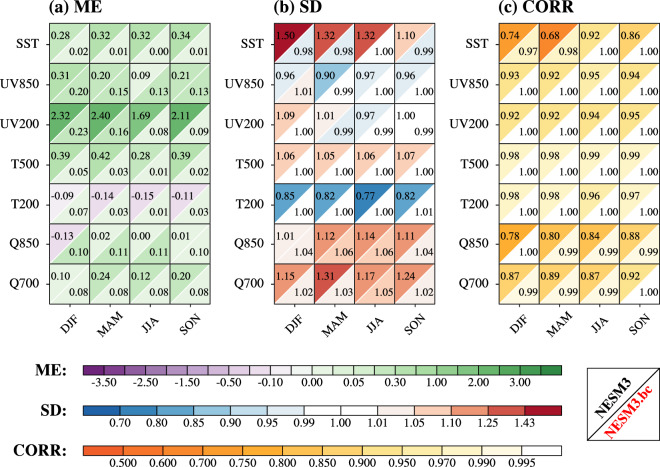
Same as Fig. 3, but for the NESM3 and the bias-corrected NESM3 (NESM3.bc) in simulating the climatological mean (1979–2014) of seasonal variability for multiple driving variables. The seasonal variability is measured by the standard deviation of the daily data across the season of one year.

We also evaluated the performance of the original and the bias-corrected NESM3 in simulating the climatological mean of the seasonal variability for the seven driving variables during the historical period from three aspects (Fig. 5). Here, we used the standard deviation of the daily data across the season in one year to measure the seasonal variability. Seasonal variability of a variable represents the amplitude of the anomalous values throughout the season, which is critical for the downscaling simulation of extreme weather. The NESM3 performance in simulating seasonal variability varies with different variables. The NSEM3 overestimates the spatial mean of SST variability throughout the year (Fig. 5a). Simultaneously, the NESM3 shows a relatively large amplitude during DJF, MAM, and JJA as well as the rather poor pattern similarity during DJF and MAM against the ERA5 (Fig. 5b and c). In terms of the wind fields, there is a relatively large overestimation of the spatial mean in the upper level throughout the year (Fig. 5a). As for the air temperature, the NESM3 overestimates the spatial mean of the 500hPa air temperature (T500) and underestimates the amplitude of the T200 in the anomalous fields throughout the year (Fig. 5a and b). In addition, the NESM3 also shows the overestimated amplitude in the low-level specific humidity throughout the year except for the 850hPa specific humidity (Q850) during DJF (Fig. 5b). For the Q850 during DJF, the NESM3 shares the relatively poor pattern similarity with the ERA5 than that of the other seasons (Fig. 5c). Taking three aspects of model performance into account, the bias-corrected NESM3 preforms greater than the original NESM3 in simulating driving variables. The bias corrections reduce the spatial mean bias of more than 17% in all variables except for UV850 during JJA as well as Q850 during MAM, JJA, and SON (Fig. 5a). In terms of the anomalous fields, the bias corrections improve the NESM3 simulation of amplitude and pattern similarity across all variables, with the ratio of the amplitude against the ERA5 between the 0.98 and 1.06 as well as the pattern similarity greater than 0.97 (Fig. 5b and c).

### Extreme values

**Fig. 6:**
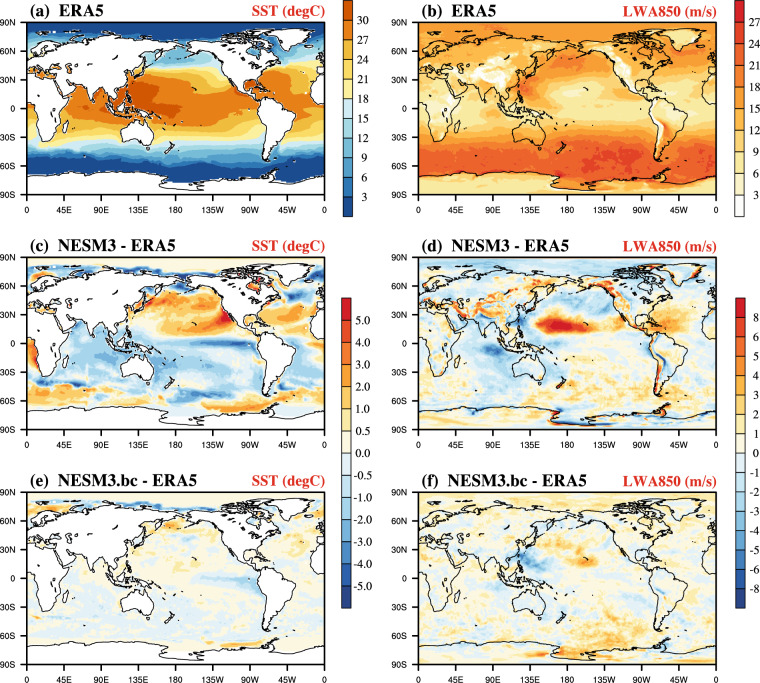
Spatial fields of the 98th percentile of the daily sea surface temperature (SST, unit: °C) and length of 850hPa wind anomaly (LWA850, unit: m/s) during July derived from 1979–2014. (**a,** **b**) the ERA5 data, (**c,** **d**) difference between the NESM3 and the ERA5, (**e,** **f**) difference between the bias-corrected NESM3 (NESM3.bc) and the ERA5.

The extreme value of the large-scale driving variable is also vital to the dynamical downscaling. Considering the SST as an important forcing in the RCM, we calculated the 98th percentile of daily SST during July from 1979–2014 for the original and the bias-corrected NESM3 as well as the ERA5 (Fig. 6a,c, and e). The original NESM3 generally shows a warm bias of more than 2 °C in the North Pacific and the west coast of Africa and a cold bias of less than −2 °C in the intertropical Pacific convergence zone, Labrador Basin, and Norwegian Sea (Fig. 6c). In the contrast, the SST extreme bias in the bias-corrected NESM3 is between -1 °C and 1 °C in almost all grid cells (Fig. 6e). In general, the overall bias of SST extreme is reduced by 68% in the bias-corrected NESM3.

In addition to the SST, the wind fields also play a vital role in transferring information into the RCM and can further affect downscaled precipitation by water vapor transport^26^. To take the direction and speed of the vector wind into consideration simultaneously, we defined the length of the vector wind anomaly (LWA) as follows:

6 $${{\rm{LWA}}}_{{\rm{i}}}=\sqrt{{({u}_{i}-\bar{u})}^{2}+{({v}_{i}-\bar{v})}^{2}}$$

where i indicates the time, and *u* (*v*) represents the zonal (meridional) wind. $\bar{u}$ ($\bar{v}$) is the climatological mean value of *u* (*v*) during the historical period. LWA can measure the deviation of the vector wind relative to its climate mean. We compared the 98th percentile of the daily length of the 850hPa wind anomaly (LWA850) between the original and the bias-corrected NESM3 (Fig. 6b,d, and f). The original NESM3 shows a obvious overestimation in the northern part of the equatorial Pacific, with a bias greater than 4 m/s (Fig. 6d). Large bias of the LWA850’s extreme (>4 m/s and <−4 m/s) also appears in the eastern part of the Central Indian Ocean and the high altitudes areas, e.g., the Tibetan Plateau and the Rocky Mountains (Fig. 6d). In contrast, the bias is greatly improved in the bias-corrected NESM3, and the overall bias of the LWA850’s extreme is reduced by 55% in the bias-corrected NESM3 (Fig. 6f).

**Fig. 7:**
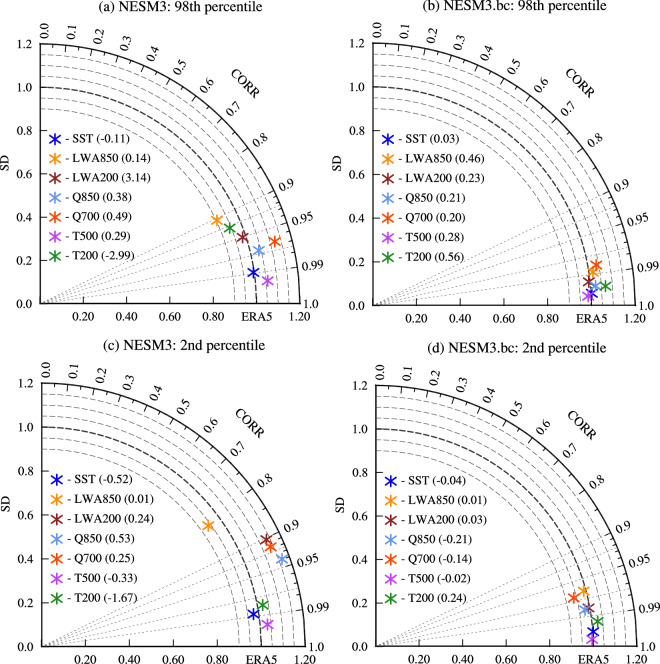
Taylor diagram for the 98th percentile and 2nd percentile of various variables during July from 1979–2014 simulated by (**a,** **c**) NESM3 and (**b,** **d**) the bias-corrected NESM3 (NESM3.bc). Various variables include sea surface temperature (SST, unit: °C), length of 850hPa wind anomaly (LWA850, unit: m/s), length of 200hPa wind anomaly (LWA200, unit: m/s), 500hPa air temperature (T500, unit: °C), 200hPa air temperature (T200, unit: °C), 850hPa specific humidity (Q850, unit: g/kg), and 700hPa specific humidity (Q700, unit: g/kg). The azimuthal position gives correlation coefficient (CORR), the radial distance from the origin indicates standard deviation (SD), and the distance between the model and the reference points provides root mean square error of the anomalous fields. Note that the SD of the model is normalized by that derived from the ERA5. The value in parentheses behind the variable name is the spatial mean bias of a variable.

Further, we comprehensively evaluated the 2nd percentile and 98th percentile of seven driving variables using daily data during July from 1979–2014 simulated by the original and the bias-corrected NESM3 using ME, SD, and CORR against the ERA5 data (Fig. 7). Among seven variables, there are relatively large amplitude biases in the anomalous field of the 98th percentile of 700hPa specific humidity (Q700) as well as the 2nd percentile of the length of the 200hPa wind anomaly (LWA200), Q700, and Q850 simulated by the NESM3 (Fig. 7a and c). Specifically, the NESM3 overestimates these variables, with a ratio of amplitude greater than 1.1 to the ERA5 data. Simultaneously, the NESM3 shows relatively poor pattern similarity in the 98th of LWA850 and T200 as well as the 2nd percentile of LWA850, LWA200, Q700, and Q850 than the other variables, characterized by CORR less than 0.95 (Fig. 7a and c). For both amplitude and pattern similarity of the anomalous fields, the bias-corrected NESM3 shows improvement in simulating the extremes of seven driving variables compared with the original NESM3, with CORR greater than 0.97 and the ratio of the amplitude between 0.94–1.06 against the ERA5 (Fig. 7b and d). In terms of the spatial mean, the bias-corrected NESM3 improves the simulation in all extreme values of seven variables against the original NESM3 by 65% on average, except for the 98th percentile of LWA850 (Fig. 7b and d). For the LWA850, the positive and negative biases of the spatial field in the original NESM3 cancel with each other (Fig. 6d), leading to a smaller spatial mean bias than that of the bias-corrected NESM3. Taking the three aspects of model performance into account, the bias corrections greatly enhance the extreme values of seven driving variables during July from 1979–2014 simulated by the NESM3.

## Usage Notes

The dataset presented in this paper was saved in NetCDF format. Many different types of software are available to manipulate or display the NetCDF data, e.g., FORTRAN, CDO, NCO, NCL, Python, Panoply, Ncview, and GrADS. More software can be found at https://www.unidata.ucar.edu/software/netcdf/software.html. The data provided here can be used to generate the initial conditions, underlying conditions, and lateral boundary conditions of the RCM.

## Data Availability

The code used to produce the bias-corrected NESM3 simulations is publicly available on the GitHub repository: https://github.com/Mengzhuo-Zhang/BC_scripts_NESM3 (last access: 21 January 2024). The code consists of an NCL (version 6.6.2, https://www.ncl.ucar.edu) script to compute non-linear trends of the variables and a few CDO (version 1.7.0, https://code.mpimet.mpg.de/projects/cdo) scripts to regrid data and correct NESM3 bias.

## References

[1] Masson-Delmotte, V. P. *et al*. *IPCC, 2021: Summary for Policymakers. In: Climate Change 2021: The Physical Science Basis. Contribution of Working Group I to the Sixth Assessment Report of the Intergovernmental Panel on Climate Change*. (Cambridge University Press, 2021).

[2] Dike, V. N., Lin, Z.-H., Wu, C. & Ibe, C. C. *Climate Impacts on Extreme Weather: Advances in weather and climate extremes*. 49–63 (Elsevier, 2022).

[3] Sanderson BM, O’Neill BC, Tebaldi C (2016). What would it take to achieve the Paris temperature targets?. Geophys. Res. Lett..

[4] Masson-Delmotte, V. P. *et al*. *IPCC, 2018: Global Warming of 1.5 °C. An IPCC Special Report on the impacts of global warming of 1.5 °C above pre-industrial levels and related global greenhouse gas emission pathways, in the context of strengthening the global response to the threat of climate change, sustainable development, and efforts to eradicate poverty*. (Cambridge University Press, 2018).

[5] Yang Y, Tang J, Wang S, Liu G (2018). Differential impacts of 1.5 and 2 °C warming on extreme events over China using statistically downscaled and bias-corrected CESM low-warming experiment. Geophys. Res. Lett..

[6] Tachiiri K, Herran DS, Su X, Kawamiya M (2019). Effect on the Earth system of realizing a 1.5 °C warming climate target after overshooting to the 2 °C level. Environ. Res. Lett..

[7] Chen Z, Zhou T, Zhang W, Li P, Zhao S (2020). Projected changes in the annual range of precipitation under stabilized 1.5 °C and 2.0 °C warming futures. Earth’s Future.

[8] Sieck K, Nam C, Bouwer LM, Rechid D, Jacob D (2021). Weather extremes over Europe under 1.5 and 2.0 °C global warming from HAPPI regional climate ensemble simulations. Earth Syst. Dyn..

[9] Lee D (2018). Impacts of half a degree additional warming on the Asian summer monsoon rainfall characteristics. Environ. Res. Lett..

[10] Aihaiti A, Jiang Z, Zhu L, Li W, You Q (2021). Risk changes of compound temperature and precipitation extremes in China under 1.5 °C and 2 °C global warming. Atmos. Res..

[11] Zhang GW, Zeng G, Yang XY, Jiang ZH (2021). Future changes in extreme high temperature over China at 1.5 °C–5 °C global warming based on CMIP6 simulations. Adv. Atmos. Sci..

[12] Sanderson BM (2017). Community Climate Simulations to assess avoided impacts in 1.5 °C and 2 °C futures. Earth Syst. Dyn..

[13] Wei Y, Yu H, Huang J, Zhou T, Zhang M, Ren Y (2019). Drylands climate response to transient and stabilized 2 °C and 1.5 °C global warming targets. Clim. Dyn..

[14] Jiang Z, Hou Q, Li T, Liang Y, Li L (2021). Divergent responses of summer precipitation in China to 1.5 °C global warming in transient and stabilized scenarios. Earth’s Future.

[15] Aerennson T, Tebaldi C, Sanderson B, Lamarque JF (2018). Changes in a suite of indicators of extreme temperature and precipitation under 1.5 and 2 degrees warming. Environ. Res. Lett..

[16] Chen H, Sun J (2019). Increased population exposure to extreme droughts in China due to 0.5 °C of additional warming. Environ. Res. Lett..

[17] Ge J (2021). Does dynamic downscaling modify the projected impacts of stabilized 1.5 °C and 2 °C warming on hot extremes over China?. Geophys. Res. Lett..

[18] Cao J, Zhao H (2020). Distinct response of Northern Hemisphere land monsoon precipitation to transient and stabilized warming scenarios. Advances in Climate Change Research.

[19] Giorgi F (1990). Simulation of regional climate using a limited area model nested in a general circulation model. J. Clim..

[20] Giorgi F, Jones C, Asrar GR (2009). Addressing climate information needs at the regional level: the CORDEX framework. WMO.Bulletin.

[21] Giorgi F, Gutowski WJ (2015). Regional dynamical downscaling and the CORDEX initiative. Annu. Rev. Environ. Resour..

[22] Wu W, Lynch AH, Rivers A (2005). Estimating the Uncertainty in a Regional Climate Model Related to Initial and Lateral Boundary Conditions. J. Clim..

[23] Plavcová E, Kyselý J (2012). Atmospheric circulation in regional climate models over Central Europe: links to surface air temperature and the influence of driving data. Clim. Dyn..

[24] Dosio A, Panitz H-J, Schubert-Frisius M, Lüthi D (2015). Dynamical downscaling of CMIP5 global circulation models over CORDEX-Africa with COSMO-CLM: Evaluation over the present climate and analysis of the added value. Clim. Dyn..

[25] Kebe I, Sylla MB, Omotosho JA, Nikiema PM, Gibba P, Giorgi F (2017). Impact of GCM boundary forcing on regional climate modeling of West African summer monsoon precipitation and circulation features. Clim. Dyn..

[26] Rocheta E, Evans JP, Sharma A (2020). Correcting lateral boundary biases in regional climate modeling: the effect of the relaxation zone. Clim. Dyn..

[27] Zhang, M.-Z., Xu, Z., Han, Y. & Guo, W. Evaluation of CMIP6 Models toward dynamical downscaling over 14 CORDEX domains. *Clim. Dyn*, 10.1007/s00382-022-06355-5 (2022).

[28] Holland, G. J., Done, J., Bruyere, C., Cooper, C. & Suzuki, A. Model investigations of the effects of climate variability and change on future Gulf of Mexico tropical cyclone activity. *Proceedings of the Offshore Technology Conference* **20690** (2010).

[29] Xu Z, Yang Z-L (2012). An improved dynamical downscaling method with GCM bias corrections and its validation with 30 years of climate simulations. J. Clim..

[30] Xu Z, Yang Z-L (2015). A new dynamical downscaling approach with GCM bias corrections and spectral nudging. J. Geophys. Res. Atmos..

[31] Piani C, Haerter JO, Coppola E (2010). Statistical bias correction for daily precipitation in regional climate models over Europe. Theor. Appl. Climatol..

[32] Colette A, Vautard R, Vrac M (2012). Regional climate downscaling with prior statistical correction of the global climate forcing. Geophys. Res. Lett..

[33] Dai AG, Rasmussen RM, Ikeda K, Liu CH (2020). A new approach to construct representative future forcing data for dynamic downscaling. Clim. Dyn..

[34] Xu Z, Han Y, Tam C-Y, Yang Z-L, Fu C (2021). Bias-corrected CMIP6 global dataset for dynamical downscaling of the historical and future climate (1979–2100). Sci. Data.

[35] Xu, Z., Han, Y., Zhang, M.-Z., Tam, F. C. Y., Yang, Z.-L., Kenawy, A. E. & Fu, C. B. Assessing the performance of a dynamical downscaling simulation driven by a bias-corrected CMIP6 dataset for Asian climate. *Adv. Atmos. Sci*, http://www.iapjournals.ac.cn/aas/en/article/doi/10.1007/s00376-023-3101-y (2024).

[36] Eyring V (2016). Overview of the Coupled Model Intercomparison Project Phase 6 (CMIP6) experimental design and organization. Geosci. Model Dev..

[37] Cao J (2021). NUIST ESM V3 Data Submission to CMIP6. Adv. Atmos. Sci..

[38] Cao J, Wang B (2019). Earth System Grid Federation.

[39] Cao J (2023). Science Data Bank.

[40] Hersbach H (2023). Copernicus Climate Change Service (C3S) Climate Data Store (CDS).

[41] Hersbach H (2023). Copernicus Climate Change Service (C3S) Climate Data Store (CDS).

[42] Hersbach H (2020). The ERA5 global reanalysis. Q. J. R. Meteorol. Soc..

[43] Tarek M, Brissette FP, Arsenault R (2020). Evaluation of the ERA5 reanalysis as a potential reference dataset for hydrological modeling over North America. Hydrol. Earth. Syst. Sci..

[44] Vogel B (2024). Evaluation of vertical transport in ERA5 and ERA-Interim reanalysis using high-altitude aircraft measurements in the Asian summer monsoon 2017. Atmos. Chem. Phys..

[45] Wu Z, Huang N (2009). Ensemble Empirical Mode Decomposition: A Noise-Assisted Data Analysis Method. Advances in Adaptive Data Analysis.

[46] Hodges JL (1958). The Significance Probability of the Smirnov Two-Sample Test. Ark. Mat..

[47] Maraun D (2015). VALUE: A framework to validate downscaling approaches for climate change studies. Earth’s Future.

[48] Xu Z, Hou Z, Han Y, Guo W (2016). A diagram for evaluating multiple aspects of model performance in simulating vector fields. Geosci. Model Dev..

[49] Xu Z, Han Y, Fu C (2017). Multivariable integrated evaluation of model performance with the vector field evaluation diagram. Geosci. Model Dev..

[50] Zhang M-Z, Xu Z, Han Y, Guo W (2021). An improved multivariable integrated evaluation method and tool (MVIETool) v1.0 for multimodel intercomparison. Geosci. Model Dev..

[51] Zhang, M.-Z., Han, Y., Xu, Z. & Guo, W. Bias-corrected NESM3 global dataset for dynamical downscaling under 1.5 °C and 2 °C global warming scenarios. *ScienceDB*, 10.57760/sciencedb.07777 (2023). 10.1038/s41597-024-03224-0 PMC11032403 38643170

